# Tagging the proteasome active site β5 causes tag specific phenotypes in yeast

**DOI:** 10.1038/s41598-020-75126-1

**Published:** 2020-10-22

**Authors:** Kenrick A. Waite, Alicia Burris, Jeroen Roelofs

**Affiliations:** 1 Department of Biochemistry and Molecular Biology, University of Kansas Medical Center, 3901 Rainbow Blvd, HLSIC 1077, Kansas City, KS USA; 2 Molecular, Cellular, and Developmental Biology Program, Division of Biology, Kansas State University, 338 Ackert Hall, Manhattan, KS 66506 USA

**Keywords:** Protein transport, Proteolysis, Proteasome, Protein quality control, Autophagy, Macroautophagy

## Abstract

The efficient and timely degradation of proteins is crucial for many cellular processes and to maintain general proteostasis. The proteasome, a complex multisubunit protease, plays a critical role in protein degradation. Therefore, it is important to understand the assembly, regulation, and localization of proteasome complexes in the cell under different conditions. Fluorescent tags are often utilized to study proteasomes. A GFP-tag on the β5 subunit, one of the core particle (CP) subunits with catalytic activity, has been shown to be incorporated into proteasomes and commonly used by the field. We report here that a tag on this subunit results in aberrant phenotypes that are not observed when several other CP subunits are tagged. These phenotypes appear in combination with other proteasome mutations and include poor growth, and, more significantly, altered 26S proteasome localization. In strains defective for autophagy, β5-GFP tagged proteasomes, unlike other CP tags, localize to granules upon nitrogen starvation. These granules are reflective of previously described proteasome storage granules but display unique properties. This suggests proteasomes with a β5-GFP tag are specifically recognized and sequestered depending on physiological conditions. In all, our data indicate the intricacy of tagging proteasomes, and possibly, large complexes in general.

## Introduction

Proteasomes are the major protease involved in the selective degradation of proteins in eukaryotic cells. As many necessary cellular processes require the timely degradation of regulators, proteasomes are essential complexes^1, 2^. Proteasomes are composed of a regulatory particle (RP) that can be sub-divided into base and lid subcomplexes, and a core particle (CP). Assembly of the 66 subunit proteasome complex is a well-regulated process involving a number of proteasome-dedicated chaperones^3^. Mutations that impair proteasome assembly or function often cause severe growth defects or even lead to cell death under conditions of proteotoxic stress. For example, deletion of the proteasome assembly chaperones Rpn14 and Nas6, or truncation of the C-terminus of the proteasome subunit Rpt5, results in poor growth at high temperatures (37 °C) or in the presence of the arginine analog canavanine^4,5^. Similarly, in mammals, several mutations that alter proteasome function are responsible for a variety of pathologies^6,7^. However, not all proteasome mutations induce these phenotypes even if they compromise function. This is because there is some functional redundancy within the proteasome, and regulatory mechanisms exists that compensate for reduced proteasome activity by upregulating proteasome levels. For instance, when the proteasome assembly chaperones Hsm3 or Nas2 are deleted individually, cells typically grow at rates similar to wild type^5,8,9^. However, yeast harboring deletions of both chaperones are very sensitive to heat and other protein folding stresses. These phenotypes are further exacerbated when the ability to upregulate transcription of proteasome subunits, as a response to a loss in proteasome activity, is eliminated. In yeast, stabilization of the transcription factor Rpn4, which itself is a proteasome substrate, results in an upregulation of proteasome levels^10–12^. In all, cells require a certain threshold of proteasome activity that is lower under conditions of optimal growth and higher under conditions that induce proteotoxic stress. Consequently, mutations like the complete deletion of many subunits are lethal under all conditions. The threshold of proteasome activity under optimal conditions is never reached. However, other mutations, such as those discussed above, are tolerated under normal conditions where proteasome activity meets a minimal threshold. Under conditions that require higher proteasome activity, these mutants fail, thus, revealing phenotypes.

Proteasomes are primarily localized to the nuclei of dividing yeast^13,14^. This localization is routinely monitored using fluorescent tags on proteasome subunits. Indeed, the localization of proteasomes has been visualized with fluorescent tags on base, lid, and CP subunits^15,16^. A number of tags on proteasome subunits have also been used to purify these complexes in a variety of organisms^17,18^. Here, we report that tagging the active site subunit β5 (Pre2) resulted in tag specific phenotypes and poor cell growth when combined with other proteasome mutations. This was surprising as this tag has been used by a number of labs, including our own, and strains expressing β5-GFP show normal incorporation of this subunit into proteasomes, and exhibit normal behavior in several microscopic and biochemical assays^19–21^. We observed the relocalization of β5-GFP tagged proteasomes to cytosolic granules under a number of conditions. Some of these were consistent with proteasome storage granules (PSGs) that form under conditions of carbon starvation^20,22,23^. However, other properties distinguish these granules from PSGs. In all, our data suggest that cells can recognize β5-GFP proteasomes as abnormal and sequester them away from the nucleus in stressful conditions.

## Results

### GFP tagging of β5 results in altered proteasome localization

**Figure 1:**
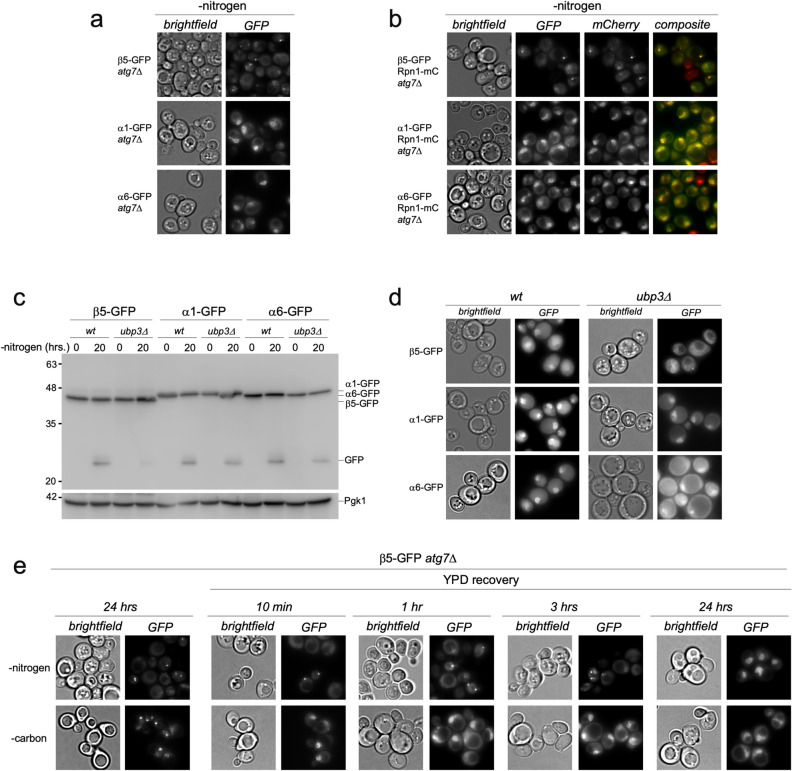
GFP tagging of β5 results in altered proteasome localization. (**a**) *atg7Δ* strains expressing β5-GFP, α1-GFP or α6-GFP from the endogenous locus were starved for nitrogen for 24 h. before images were collected. (**b**) *atg7Δ* strains expressing Rpn1-mCherry in combination with either β5-GFP, α1-GFP or α6-GFP, were starved for nitrogen for 24 h. and imaged as in (**a**). (**c**) WT or *ubp3Δ* yeast expressing β5-GFP, α1-GFP or α6-GFP were starved for nitrogen for 24 h. Protein lysates were obtained using the alkaline lysis method, separated on SDS-PAGE, and immuno-blotted for GFP, and PGK1 (loading control). (**d**) WT or *ubp3Δ* yeast with β5-GFP, α1-GFP or α6-GFP tags were starved as in (**a**) and microscopy was performed. (**e**) β5-GFP *atg7Δ* yeast were starved for nitrogen or carbon for 24 h. Microscopy shows the β5-GFP signal largely in cytosolic granules. For recovery, cells from 10 mL of culture were harvested and transferred to 10 mL of rich media (YPD). Microscopy was performed at indicated times.

We have previously reported that autophagy of the CP and the RP occur through different pathways, a model that has been supported by subsequent data^19,24,25^. Our initial model was, in part, based on subcomplex localization in autophagy mutants; RP remained largely nuclear in strains deleted for ATG7 or ATG17, while CP appeared in cytosolic punctate structures^19^. These phenotypes were apparent when the β5 subunit of the proteasome CP was tagged with GFP^19^ (Fig. 1a, top panel). Unexpectedly, we observed little to no granules in *atg7Δ* strains where the CP subunit α1 or α6 was fused with GFP (Fig. 1a). To test if the phenotype was associated with tagging of β subunits or active site subunits specifically, we next tagged the other active site subunits, β1 and β2, as well as the non-catalytic subunit β4. The β1 and β2 active site subunits showed no granule formation in *atg7Δ* strains starved for nitrogen (supplementary Fig. S1), indicating this phenomenon was not specifically associated with active subunits. We did, however, observe granule formation with GFP-tagged β4, (supplementary Fig. S1). Considering β4 is next to β5 in the half CP^26^, this might indicate that this area of CP is particularly sensitive to tags, potentially due to conformational changes associated with efficient catalysis (see below and supplementary Fig. S2). α1-,α6- and β5-GFP tagged strains all showed normal autophagy upon nitrogen starvation in a wildtype background as observed by free GFP formation on immunoblots (Fig. 1c). Furthermore, microscopic analyses showed vacuolar localized fluorescence indicative of autophagic degradation (Fig. 1d). Thus, the difference in localization we observed in the *atg7Δ* background likely resulted from a tag-induced artifact under this specific condition for the GFP-tag on the β5 subunit. To further investigate this, we generated strains where we combined the different GFP tagged CP subunits in the *atg7Δ* background with the mCherry-tagged regulatory particle subunit Rpn1. The Rpn1-mCherry signal remains nuclear in *atg7Δ* cells that are starved for nitrogen and the presence of α1-GFP or α6-GFP did not change this localization (Fig. 1b). However, the presence of β5-GFP induced a relocalization of Rpn1-mCherry. As opposed to nuclear localization, Rpn1-mCherry co-localized with β5-GFP positive cytosolic puncta upon nitrogen starvation of these *atg7*Δ cells (Fig. 1b). This was not unique for Rpn1-mCherry and was the direct result of tagging β5 as tagging this subunit changed the localization of α1, Rpn5 and Rpn2 under this condition as well (supplementary Fig. S1). Our data show that all of these tagged subunits efficiently incorporate into active proteasomes with no obvious assembly defects (supplementary Fig. S2). In all this shows that α1-GFP, α6-GFP, β1-GFP, and β2-GFP, represent physiological CP localization upon nitrogen starvation when autophagy is disrupted. The C-terminal tagging of β5 not only changed the localization of CP, but also RP (as confirmed with base and lid tags), suggesting the tag caused a relocalization of proteasome holo-enzymes under our assay conditions.

We previously reported that nitrogen starvation-induced autophagy of the CP, as monitored with a β5-GFP tag, was reduced upon deletion of *UBP3*^19^. Surprisingly, we did not observe this to the same extent when using α1-GFP or α6-GFP to monitor CP as evident from the higher levels of free GFP detected on immunoblots for the latter two (Fig. 1c, compare lane 4, 8, and 12). Consistently, we observed substantial vacuolar fluorescence after nitrogen starvation for α1-GFP *ubp3Δ* and α6-GFP *ubp3Δ* strains upon microscopic analyses, while the vacuolar fluorescence for nitrogen starved β5-GFP *ubp3Δ* was strongly reduced (Fig. 1d). In all, our data indicate that Ubp3 is not as important for normal CP autophagy as we initially concluded. Nevertheless, we did observe some reduction in free GFP derived from α1-GFP or α6-GFP upon *UBP3* deletion (Fig. 1c).This suggests that Ubp3 does play a modest role in CP autophagy, although we cannot exclude a minor defect in lysosomal targeting resulting from tag-induced effects. Consistent with this, Ubp3 appears to be important for proteasome autophagy under specific conditions including carbon starvation of *blm10*Δ cells^25^. In our hands, the GFP tag on β5 appears to increase proteasome dependence on Ubp3 for autophagy.

Proteasomes relocalize from cell nuclei to cytosolic puncta termed proteasome storage granules (PSGs) under conditions of carbon limitation^22^. To determine if the β5-GFP puncta had properties similar to proteasome storage granules (PSGs), we monitored their dynamics following a switch from minimal media lacking carbon or nitrogen to rich media (yeast extract, peptone, dextrose (YPD)). Upon reintroduction of carbon, PSGs dissipate within minutes and proteasomes re-localize to the nucleus quickly^14,20,21,27^. Under carbon starvation, our β5-GFP strain showed similar PSG dynamics, indicating this tag does not induce different behaviors for proteasomes in our strain background or with our assay conditions. Thus, β5-GFP granules that form under carbon starvation behave as normal PSGs and likely represent physiological granules (Fig. 1e). Unlike PSGs, granules induced upon nitrogen starvation in the β5-GFP tagged *atg7Δ* strain persisted up to 3 h following transfer to rich media (Fig. 1e). Thus, the granules that form in the *atg7*Δ strain under nitrogen starvation are distinct from PSGs and are putatively regulated by different factors.

### GFP tagging of β5 results in less CP activity than untagged proteasomes

**Figure 2:**
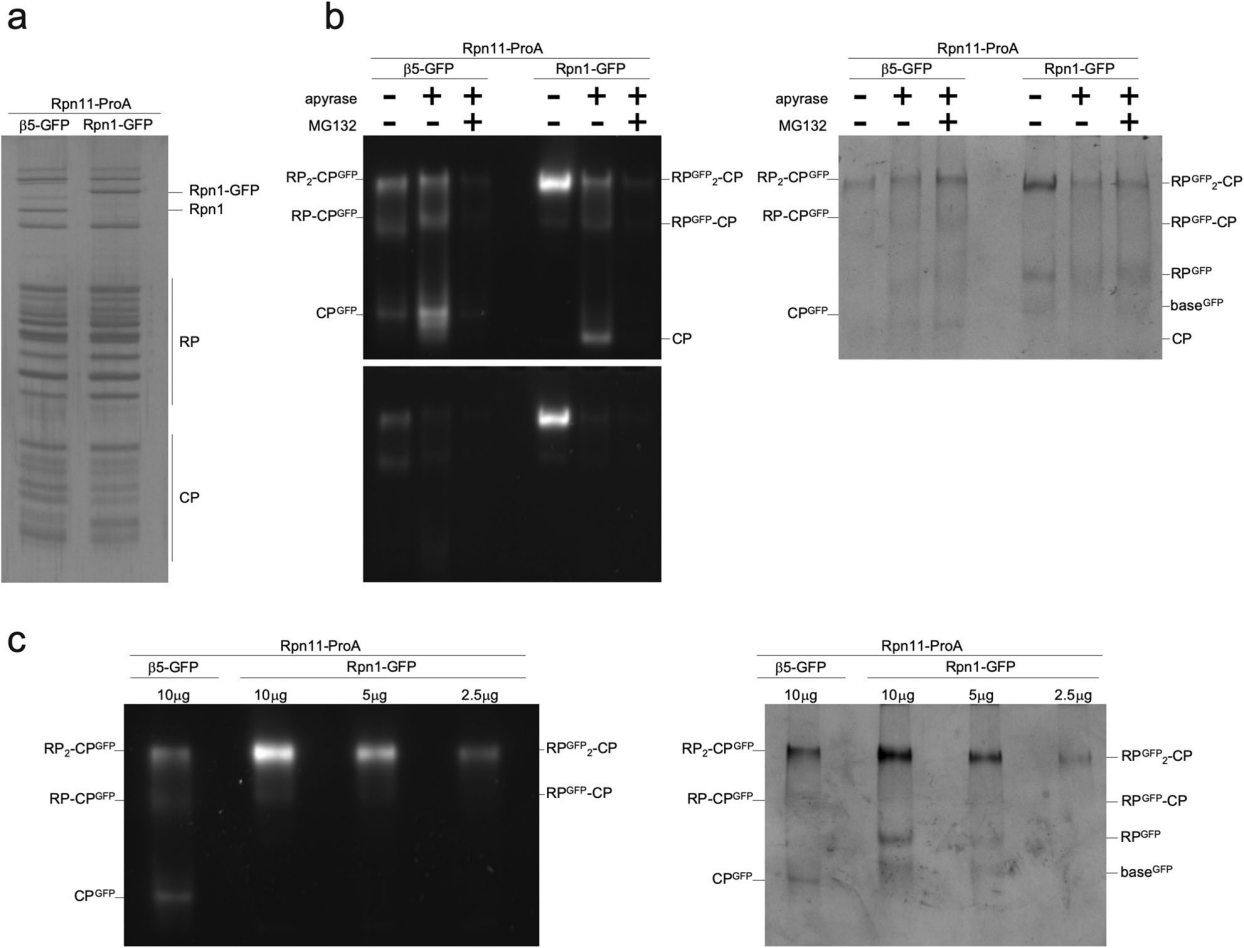
β5-GFP tagged proteasomes have reduced proteolytic activity. (**a**) Yeast strains with indicated tags were grown in YPD overnight. Cells were lysed and the protein A affinity purification tag on Rpn11 (Rpn11-ProA) enabled proteasome purification with IgG affinity resin. After elution, samples were separated by SDS-PAGE and gel stained using Coomassie brilliant blue (CBB). (**b**) Proteasomes purified in (**a**) were treated with apyrase (to deplete ATP and ADP) in the presence or absence of the proteasome inhibitor MG132. DMSO was used as a vehicle control (−). Samples were separated by native gels and peptidase activity was visualized using LLVY-AMC in gel activity assay in the presence (top) or absence of 0.02% SDS (to visualize free CP) and stained with CBB (right). (**c**) A sample of 10 μg β5-GFP purified proteasomes was compared to a dilution series of Rpn1-GFP purified proteasomes using native gel analysis and visualized using LLVY-AMC activity in the presence of 002% SDS (left) and CBB (right).

**Figure 3:**
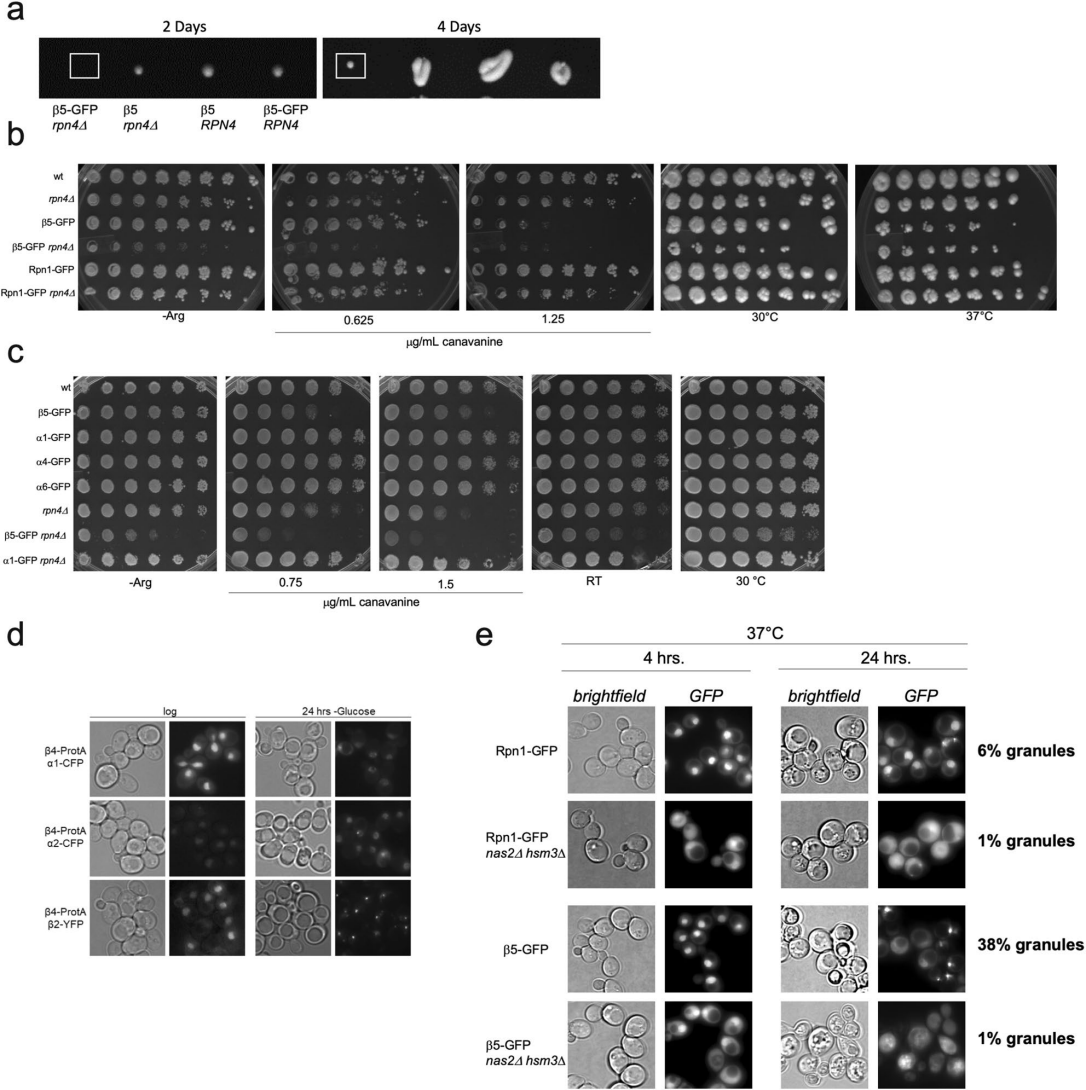
GFP tagging of β5 results in growth phenotypes in yeast. (**a**) Colonies derived from a tetrad dissection where one spore contained both the β5-GFP tag together with a deletion of RPN4 (indicated with square). Images from plate 2 days (left) and 4 days (right) post dissection. The dramatically slower growth was only observed for β5-GFP *rpn4*Δ (n = 4). Note changed colony shape for 2nd to 4th colonies was caused by re-streaking. (**b**) Indicated strains were grown to log phase in rich media. The equivalent of 1 mL of cells at OD_600_ 1 was harvested, washed and resuspended in sterile water. Fourfold serial dilutions were transferred onto YPD plates, plates lacking arginine, or lacking arginine and containing indicated concentration of canavanine using a pin array. Plates were grown at 30 °C or the indicated temperature. (**c**) as (**b**) for indicated strains and conditions. (**d**) β4-protein A tagged cells expressing α1-CFP, α2-CFP or β2-YFP were grown to log phase in YPD media, then switched to carbon starvation media. Microscopy was performed at indicated times. (**e**) WT and NAS2 HSM3 double deletion strains expressing Rpn1-GFP or β5-GFP were grown at 37 °C in YPD for indicated time and analyzed by microscopy. Quantification of the percentage of cells with granules and percent unbudded were performed manually using FIJI**.**

Although both CP and RP are present in PSGs, these subcomplexes are not proposed to be associated as they can be targeted to these structures independently^21,22,25^. Our data suggest that the whole 26S proteasome is present in the β5-GFP induced granules, as the β5-GFP tag caused RP to colocalize in those granules (Fig. 1b). Therefore, we reasoned that there were likely structural differences present in the 26S complex when β5 is tagged. To test this, we generated yeast expressing Rpn1-GFP or β5-GFP with a protein-A tag on Rpn11 for affinity purifications. When we purified these proteasomes from rich media, we did not detect any alterations in proteasome composition, indicating that all proteasome subunits were incorporated regardless of the subunit tagged (Fig. 2a). This is consistent with our previous analyses showing that these tagged subunits are efficiently incorporated into 26S proteasomes^19^. However, one possible explanation for translocation of 26S complexes in doubly tagged strains involves artificial 26S stabilization through the β5-GFP tag. Therefore, to test for differences in stability between 26S complexes, we removed nucleotide from the proteasome purifications using the enzyme apyrase in the presence or absence of the proteasome inhibitor MG132 (Fig. 2b). 26S proteasomes are unstable in the absence of ATP or ADP and proteasome inhibitors can stabilize 26S complexes in the absence of these nucleotides^28^. However, both β5-GFP and Rpn1-GFP tagged proteasome displayed RP-CP dissociation upon ATP depletion as reflected by the increased amount of free CP (Fig. 2b lanes 2 and 5). Thus, it does not appear that the β5-GFP proteasomes are more stable than Rpn1-GFP proteasomes. However, the data did seem to indicate a difference in proteasome activity. To pursue this further we analyzed whole cell lysate where β5-GFP tagged CP appeared to be less active that untagged CP (supplementary Fig. S2). One caveat here is that Rpn4 dependent upregulation of proteasomes might complicate the analysis, particularly because we observed a synergistic effect of combining β5-GFP and a deletion of RPN4 (Fig. 3a,b). While purifications have the disadvantage of introducing an additional tag, upon purification, this is removed by Tev protease cleavage and we can ensure equal proteasome amounts. . We tested the relative activity of these purified proteasomes by comparing the activity present in β5-GFP proteasomes to a dilution series of purified Rpn1-GFP proteasomes (Fig. 2c). Here, we observed that, indeed, β5-GFP proteasomes are less active than Rpn1-GFP proteasomes; 5 μg of purified Rpn1-GFP material appeared to contain an amount of 26S proteasomes equal to the β5-GFP containing prep based on the Coomassie stained gel; however, the activity of the β5-GFP proteasomes was reduced about twofold as it resembled the activity of 2.5 μg of the Rpn1-GFP proteasome preparation. Thus, the β5-GFP tag likely interferes with the catalytic activity of this subunit. In sum, it appears that β5-GFP tagged proteasomes are less active than proteasomes with an untagged β5 .

### GFP tagging of β5 results in growth phenotypes in yeast

Phenotypically, reduced proteolytic activity commonly presents as sensitivity to high temperature or the arginine analog canavanine, as these conditions induce proteotoxic stress. However, these phenotypes are not always apparent due to upregulation of proteasomes by the transcription factor Rpn4. Rpn4 levels increase under conditions of reduced proteasome activity, because Rpn4 is also a proteasome substrate. As a result, any compromised proteasome function resulting from mutated subunits is often masked by Rpn4-dependent transcriptional upregulation of proteasome genes^8,10,11^. Our efforts to generate a β5-GFP tagged strain, also harboring a deletion of *RPN4,* using standard yeast transformation methods were not successful. On the other hand, a deletion of *RPN4* was readily obtained in the Rpn1-GFP background. To test if this indicated a synthetic lethality between β5-GFP and *rpn4*Δ, we generated a diploid strain harboring β5-GFP and *rpn4Δ* single mutations. While tetrad dissections of this diploid showed that cells with both β5-GFP and a deletion of *RPN4* were viable, we did observe very slow growth, exclusively for those cells (Fig. 3a, colony on the left). Next, we tested the growth of the single and double mutants under conditions of proteolytic stress. Strains deleted for *RPN4* are known to show slow growth and some sensitivity to proteolytic stress^12,29^, which was exacerbated by the β5-GFP tag (Fig. 3b). Furthermore, β5-GFP alone caused growth defects in the presence of high levels of canavanine. These phenotypes were specific to the β5-GFP tagged strains, as they were not observed with the Rpn1-GFP tag. This was consistent with our observations of reduced proteasome activity for β5-GFP tagged proteasomes (Fig. 2c).

To test if these properties were associated with any other tagged CP subunits or specifically associated with β5, we compared growth under these conditions for GFP tagged β5, α1, α4, or α6. We did not observe any compromised growth when strains contained C-terminal GFP tags on subunits α1, α4, or α6. This shows that the tagging the α1, α4, or α6 CP subunits did not lead to any obvious defects in proteasome function. Indeed, even in a *rpn4*Δ background, α1-GFP showed no increased sensitivity (Fig. 3c). However, when we performed tetrad dissections to combine these different fluorescently tagged CP subunits with a C-terminal truncation of Rpn11 which has similarities to the Rpn11 mutation found in *mpr1-1* or *rpn11-m1* strains^30,31^, we observed synthetic lethality for the Rpn11 truncation with GFP-tagged α1, α2, and β5 (not shown). Only the combination of Rpn11 truncation with α4-GFP was well tolerated by cells. With respect to localization, we have observed altered behavior when either α1 or α2 were tagged with CFP in combination with a protein A tag on β4. As mentioned previously, proteasomes exit the nucleus and localize to PSGs in the cytoplasm during carbon starvation; however, strains with an α1- or α2-CFP tag, in combination with a β4 protein A tag, failed to form granules following 24 h of starvation (Fig. 3d). Like β5-GFP, β4-GFP caused mislocalization of proteasomes in an ATG7 deleted background following nitrogen starvation. This combined with the observation that β4-proA affects granule formation under carbon starvation supports the idea of this area of the CP being particulary sensitive to tagging. Interestingly, proteasomes with a β4 protein A tag in combination with β2-YFP formed granules similarly to what has previously been described, suggesting β4 protein A alone may not be responsible for failure in granule formation upon carbon starvation but more of cumulative effect of where the proteasome is tagged. In all, it appears that certain combinations of tagged or mutant proteasome subunits can affect both cell viability and proteasome localization following certain stress conditions. More specifically, the commonly used GFP-tag on the active site subunit β5 in yeast results in phenotypes typically observed in strains with compromised proteasome function.

Besides these various mutation combinations and disruption of *RPN4*, proteasome chaperone mutations are also known to affect cell viability under conditions of protein stress^4,8,9^. To determine if a GFP tag on β5 or Rpn1 would exacerbate these phenotypes, we generated strains harboring a deletion of the regulatory particle chaperones *NAS2* and *HSM3* in a background expressing Rpn1-GFP or β5-GFP. Under logarithmic growth at 37 °C, the majority of GFP fluorescence in the WT Rpn1-GFP and β5-GFP strains was nuclear (Fig. 3e). Upon deletion of NAS2 and HSM3, the fluorescent signal in cells appears more diffuse and cytoplasmic for both Rpn1-GFP and β5-GFP, perhaps suggesting that interfering with proteasome assembly reduces the efficiency of proteasome nuclear import. Indeed, it has been shown that excess Rpt subunits were sequestered into cytosolic puncta when proteasome assembly was compromised^9^. The cytosolic enrichment of GFP in our NAS2 and HSM3 deletion mutants might result from Rpn1 and β5 being incorporated into proteasomes at reduced rates. When these strains were grown for 24 h at 37 °C, to induce proteolytic stress, there was no apparent growth defect resulting from the introduction of the Rpn1-GFP tag. The *nas2*Δ *hsm3*Δ cells with β5-GFP, on the other hand, appeared to be dying (see below). Microscopic analyses showed that for the Rpn1-GFP tagged strains, localization remained largely the same at both 4 h and 24 h of growth at 37° C. For the strain lacking *NAS2* and *HSM3*, both timepoints showed less nuclear and more cytoplasmic fluorescence. In contrast, 24 h of growth at 37° C showed significant differences for the β5-GFP fluorescent signal. In the wildtype background, β5-GFP relocated from the nucleus to cytosolic puncta. Here, 38% of cells with β5-GFP tagged formed granules compared to 6% for Rpn1-GFP tagged cells. These granules appear similar to those observed under nitrogen starvation in a β5-GFP *atg7Δ* strain. In the β5-GFP tagged *hsm3*Δ *nas2*Δ strain, the fluorescence signal was close to background levels, and interestingly, the majority of cells failed to complete budding (97% were unbudded compared to only 10% in Rpn1-GFP tagged *hsm3*Δ *nas2*Δ). Many cells also appeared to be dead or dying. In summary, mutations that compromise proteasome levels and cell viability under stress are exacerbated when combined with a β5-GFP tag.

### Properties of β5-GFP granule formation

**Figure 4:**
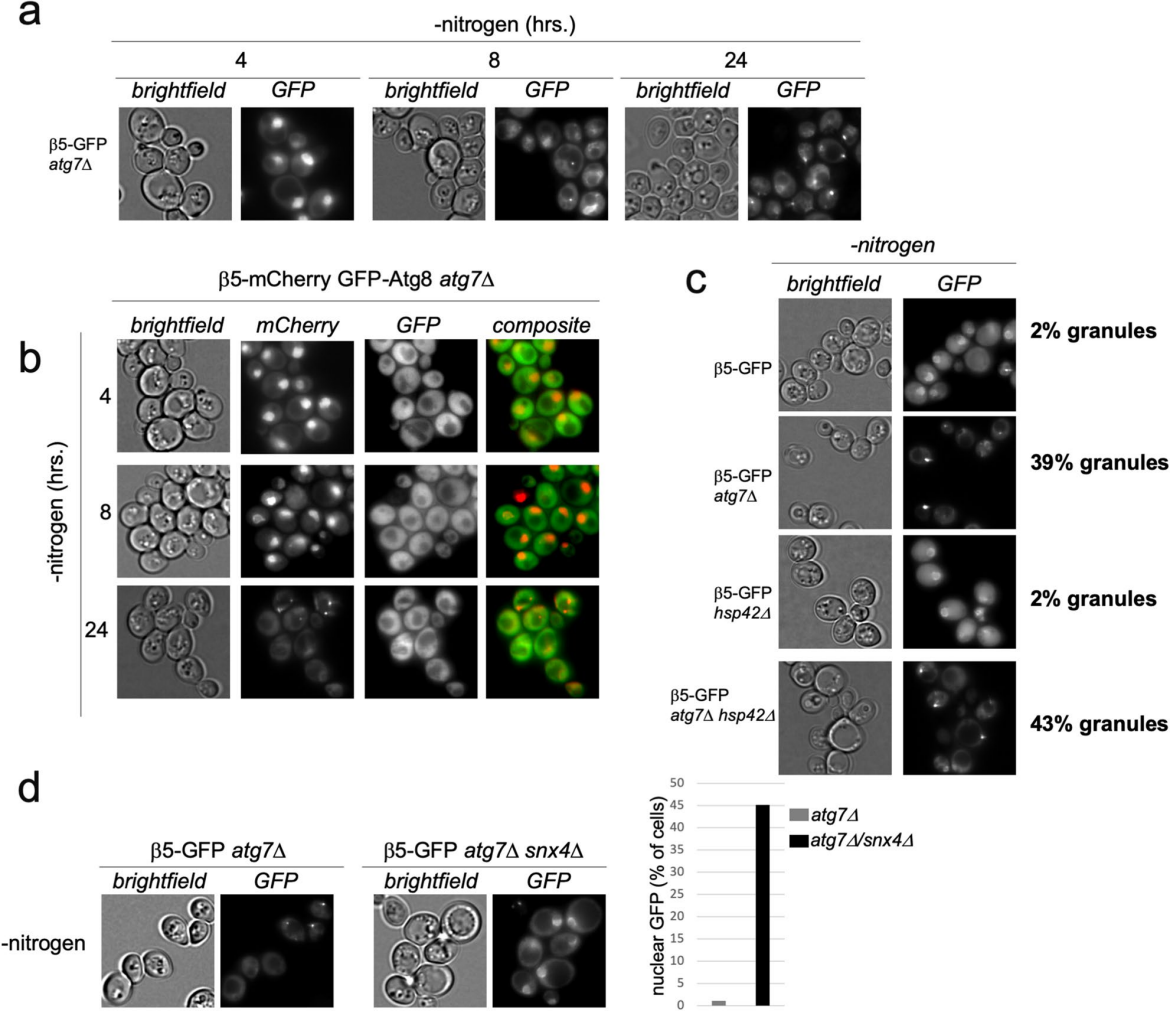
Properties of β5-GFP granule formation. (**a**) *atg7Δ* strains expressing β5-GFP were starved for nitrogen as in Fig. 1a and analyzed microscopically at indicated times. (**b**) *atg7Δ* yeast expressing β5-mCherry and GFP-Atg8 were starved for nitrogen and analyzed by microscopy at the indicated times. (**c**) WT, *atg7Δ*, *hsp42Δ* and *atg7Δhsp42Δ* yeast expressing β5-GFP were starved for nitrogen as above or grown in rich media at 37 °C for 24 h. Quantification of the percentage of cells with granules was performed manually using FIJI. (**d**) β5-GFP expressing yeast with *atg7Δ* or *atg7Δsnx4Δ* were starved for nitrogen for 24 h. Quantification of number of cells with nuclear GFP was carried out using FIJI.

As the β5-GFP granules formed upon nitrogen starvation in strains that were defective in autophagy (by a deletion of ATG7, ATG17, or UBP3), we reasoned these granules might represent stalled autophagic cargo. To analyze this, we first monitored how quickly β5-GFP granules formed upon nitrogen starvation in β5-GFP *atg7Δ* cells. Granule formation was relatively slow as we did not detect granules at 4 h (Fig. 4a). At 8 h we detected granules, but they were not as abundant or intense as compared to 24 h post starvation initiation. Autophagosomes, on the other hand, form within minutes following starvation and autophagic bodies can be detected as early as 2 h inside vacuoles^32,33^. Furthermore, Atg8, a protein found in autophagosomes, did not accumulate in the granules we observed (Fig. 4b). The latter was consistent with the published literature as Atg7 is required in this process^34,35^. Thus, β5-GFP specific granules that formed after nitrogen starvation in strains defective for autophagy are likely not failed cargo since autophagosomes cannot form in this condition. Instead, the β5-GFP granules could be similar to granules that form upon proteasome inhibition and encompass aggregated proteasomes^36^. As this pathway depends on Hsp42, we deleted HSP42 in the β5-GFP *atg7Δ* background and monitored granule formation (Fig. 4c). Since we observed no difference in the appearance or amount of β5-GFP granules that formed following nitrogen starvation, the β5-GFP granules that formed following nitrogen starvation do not represent an intermediate in the Hsp42-dependent pathway of inactive proteasome autophagy.

Next, we examined the role of Snx4/Atg24, a sorting nexin that plays a role in proteasome autophagy upon nitrogen starvation^24^. Interestingly, α4-GFP *atg8*Δ cells used by Nemec et al. formed cytoplasmic punctate structures similar to our observations with a β5-GFP *atg7Δ* strain. These granular structures required the presence of Snx4, which led to the model that Snx4 triggered proteasome cytosolic agglomeration prior to autophagic engulfment. When we compared nitrogen starved β5-GFP *atg7Δ* with β5-GFP *atg7Δ snx4Δ* cells, we noticed that deletion of SNX4 largely prevents granular structures and the fluorescent signal remained predominantly nuclear (Fig. 4d). Considering the role of Snx4 in proteasome autophagy, these β5-GFP granules are possibly stalled autophagy cargo. These cargo granules must be a step prior to autophagosome formation because they can be observed in ATG7 and ATG17 deleted strains, which do not form autophagosomes. While these granules are not associated with autophagosomes, they perhaps are a prerequisite for proteasome CP autophagy. Another possibility is that these proteasomes are recognized as faulty, and Snx4 facilitates their cytosolic retention in granules. This seems more likely since other proteasome GFP reporters remained nuclear when autophagy was blocked. That said, Snx4 is also required for autophagy of proteasomes when the tag does not induce granular localization upon autophagy impairment. Here, Rpn2 and Rpn5 were not reported to be in cytosolic granules prior to autophagy, but required Snx4 for autophagic degradation^24^. In all, it appears that β5-GFP proteasomes are regulated by different machinery in yeast than proteasomes with other subunits tagged due to a synthetic effect of the tag under these specific conditions.

## Discussion

Efforts by several labs in recent years have revealed a surprising complexity in the localization of proteasomes under different stress conditions in a number of organisms^9,19,20,24,25,37,38^. Monitoring the dynamic nature of proteasome assembly, proteasome autophagy, and PSG formation has relied on fluorescent tags fused to particular subunits. However, due to its complex structure, numerous conformations, and the many regulators that bind the complex under different conditions^1^, particular care should be taken in deciding which proteasome subunits should be tagged and where. For instance, the C-terminal regions of the six base Rpt subunits should never be tagged as these C-termini are involved in crucial interactions with CP^4,39–41^. We have found that tagging the β5 subunits of the proteasome core particle, which has been used in several studies and often appears fully functional compared with untagged β5, altered 26S proteasome localization under specific conditions of cellular stress. We report that not only does this tag cause mis-localization of 26S proteasomes, but that proteasomes tagged at this subunit have reduced proteolytic activity compared to proteasomes that are tagged on the RP subunit Rpn1.

Considering that the β5 subunit contains the active site residues responsible for a major part of the proteolytic activity of the CP^42^, the reduced activity might not be completely surprising. To become proteolytically active, β5 needs to undergo autocatalytic pro-peptide processing. If this is compromised it could result in less chymotrypsin-like activity and an accumulation of a higher molecular weight β5-GFP on immunoblots of total lysate or purified proteasomes due to the presence of propeptide. While we observed a higher molecular weight species on western blots when blotting nitrogen starved cells for GFP (supplementary Fig. S4, black arrow), we did not detect β5-propeptide associated with these bands when using a specific antibody. We however cannot exclude the possibility that the pro-peptide form is below our detection limit under these assay conditions. Another possibility that may explain the observed reduction in activity is that tagging β5 with GFP results in less efficient CP assembly. While this could explain the growth phenotypes we observed, it would not explain the reduced activity for assembled purified proteasomes. The β5 active site is located on the inner surface of the barrel shaped CP, while the C-terminus, where GFP is fused, is outfacing. Based on the general thought that CP does not undergo dramatic conformational changes during the hydrolytic process, GFP tagging might not be expected to compromise function as long as it does not interfere with the ability of CP to interact with other complexes. As the latter does not appear to be the case, at least for the ability to bind RP, the tagging of β5 might impact subtle conformational changes that do occur in CP. For example, the engagement of the active site with an inhibitor is known to cause allosteric changes at the CP alpha ring where RP binds^28,43^. If the additional mass at the C-terminus of β5 (or β4) changes such dynamics, or changes relative abundance of the different forms of the 26S proteasome that have been observed^44^, it might change the fate of 26S complexes, uniquely in this background, under particular conditions. Such a subtle change might explain why β5 acts similar to other tagged proteasomes in one condition and is uniquely recognized in others. The regulation and localization of β5-tagged proteasomes described here may expose a pathway that recognizes aberrant or inactive proteasomes under particular conditions.

In conclusion, our data show that tags that appear innocuous based on several analyses can still interfere with particular functions. We think this is particularly the case for hubs of protein–protein interactions where different factors might associate with a complex under different conditions. Furthermore, it is important to realize that complexes with critical function in the cell are likely to have compensatory mechanisms in place that might be activated in tagged strains where detrimental effects of a tag may be masked. Thus, for important multi-subunit complexes, the use of a number of tags on different subunits might be critical. For the proteasome specifically, we did not observe tag induced phenotypes for the various assays we conducted with α1, α4,, α6, β1, or β2-GFP tagged CP as reporters. However, depending on the research question being addressed, tags that function well in one condition might not be ideal in another, in which case it is important to use multiple tags to show the same effects.

## Materials and methods

### Yeast strains

All strains used in this study are reported in supplementary table S1. Our background strains are DF5 derived haploids SUB61(Matα) or Sub62(MatA); (*lys2-801 leu2-3, 2-112 ura3-52 his3-Δ200 trp1-1*)^45^. Standard PCR based procedures (primers and plasmids presented in supplementary table S2) were used to delete specific genes from the genome or introduce sequence at the endogenous locus that resulted in the expression of C-terminal fusions of GFP, mCherry, or CFP^46–48^. Yeast dissections were performed using standard practices and desired strains were confirmed by drug selection as well as PCR based methods. GFP-atg8 expressing strains were generated by transformation with integrating BS-Ura3-GFP-Atg8, a gift from Zhiping Xie (Addgene plasmid #69194)^49^.

### Yeast growth conditions

Overnight cultures of yeast were diluted to an OD_600_ of 0.5 and grown in yeast peptone dextrose (YPD) medium to an OD_600_ 1.5 (approximately 4 h). To induce starvation, cultures growing logarithmically were centrifuged, washed with the respective starvation medium, re-inoculated at an OD_600_ of 1.5, and incubated at 30 °C with constant shaking. 37 °C cultures were grown in YPD medium. For proteasome purifications, 1.5 L of YPD were used to grow strains with a protein A tag on Rpn11. Cultures were harvested by centrifugation, washed with sterile water and frozen at − 80 °C until proteasome purifications were performed. Yeast phenotypes were assayed by growing cultures to log phase and harvesting 1 OD_600_. Pellets were washed and resuspended in sterile water. This suspension was serially diluted (fourfold), stamped to assay plates, and incubated at 30 °C unless otherwise indicated.

### Protein lysates and electrophoresis

For western blotting, 2 OD_600_ of cells were collected at indicated times following starvation and stored at − 80 °C. The alkali lysis method was used as previously described, and 10 μL of lysate was loaded on SDS-PAGE^50^. Following electrophoreses, samples were transferred to PVDF membranes and immuno-blotted using antibodies against GFP (1:500; Roche, #11814460001), Pgk1 (1:10,000; Invitrogen, #459250) and β5-propeptide (1:2000, a generous gift from Dr. John Hanna at Harvard Medical School) followed by incubation with horseradish-peroxidase conjugated :secondary antibodies. Horseradish-peroxidase activity was visualized using the Immobilon Forte Western HRP substrate (Millipore) and images were acquired sing the G-box imaging system (Syngene) with GeneSnap or GeneSys software (version 1.7.2., https://www.syngene.com/). Data shown are representative of consistently observed trends from at least three independent biological replicates.

For native gel analyses, proteasomes were affinity purified using IgG resin as previously described^18,51^. Equal protein was loaded on gel and separated by electrophoresis (90 V, 2.5–3 h, 4 °C). The gels were then incubated with suc-LLVY-AMC (100 μg/mL) and imaged using a Syngene G-Box (UV excitation, 440 nm band-pass emission filter) to visualize peptidase activity of proteasome complexes. Samples for SDS-PAGE were prepared by mixing equal protein with 1/5 volume of 6X SDS sample buffer (10% SDS, 40% glycerol, 60 mM DTT, 345 mM Tris–HCl [ph. 6.8] 0.005% bromophenol blue). Apyrase treatment of these purified proteasomes was performed first by diluting samples in proteasome lysis buffer (50 mM Tris (pH 8.0), 5 mM MgCl_2_, 1 mM ATP, 1 mM EDTA), then adding 30 milliunits/μL apyrase and incubating for 45 min at 30 °C. Proteasome inhibitor (10 μM MG132) or DMSO as a control were added at the same time as apyrase. Samples were then run on native gels and visualized as described above. To monitor native proteasome composition and activity from whole cell lysate, the cryogrinding method was used as previously describe^51^. Briefly, 50 OD_600_ of yeast cultures were collected, washed, and frozen dropwise in liquid nitrogen. These pellets were ground using mortar and pestle in the presence of liquid nitrogen then resuspended in lysis buffer (50 mM Tris (pH 7.5), 5 mM MgCl_2_, 1 mM ATP, 1 mM EDTA). Equal volumes of lysate were loaded on native gels. Following electrophoreses, native gels were imaged for GFP (excitation with blue LED and 525 nm long pass emission filter) and mCherry (with green LED and 605 nm long pass emission filter) using G:Box mini from Syngene. The gels were then incubated with suc-LLVY-AMC (100 μg/mL) and imaged on the G:Box mini to visualize peptidase activity of proteasome complexes (UV excitation, 440 nm long pass emission filter).

### Fluorescence microscopy

All microscopy was done with live yeast cells where proteasome subunits were fluorescently tagged (Rpn1-GFP, Rpn5-GFP, Rpn2-mCherry, α1-GFP, α6-GFP, β1-GFP, β2-GFP, β4-GFP, β5-GFP, or β5-mCherry) at their endogenous locus with expression driven by the endogenous promotor. After indicated treatments, approximately 2 ODs of cells were pelleted, washed with PBS, then resuspend in 30 μL of PBS, and 3 μL mounted on 1% soft agar slides as described by Muller^52^ (https://www.youtube.com/watch?v=ZrZVbFg9NE8). All imaging by fluorescence microscopy was done within 10 min following the wash step to avoid changes in localization due to prolonged incubation on slides. Images were acquired at room temperature using a Nikon Eclipse TE2000-S microscope at 600 × magnification with a Plan Apo 60 ×/1.40 objective equipped with a Retiga R3 camera (Q-Imaging). Images were collected using Metamorph software (Molecular Devices) and analyzed using FIJI.

## Supplementary information

Supplementary Information.

## Abbreviations

CP: Core particle
RP: Regulatory particle
