# Supplementary material for: Tagging the proteasome active site β5 causes tag specific phenotypes in yeast
Source: Sci Rep. 2020 Oct 22;10:18133. doi: 10.1038/s41598-020-75126-1 (PMC7582879; doi:10.1038/s41598-020-75126-1)
Supplement: Supplementary file 1 — Supplementary Information. [file 41598_2020_75126_MOESM1_ESM.pdf]

Supplementary materials for

Tagging the proteasome active site ß5 causes tag specific phenotypes in yeast

Kenrick A. Waite[1], Alicia Burris[1,2] and Jeroen Roelofs[1]*

[1] Department of Biochemistry and Molecular Biology, University of Kansas Medical Center, Kansas City, 3901 Rainbow Blvd, HLSIC 1077, Kansas, USA
[2] Molecular, Cellular, and Developmental Biology Program, Division of Biology, Kansas State University, 338 Ackert Hall, Manhattan, KS 66506 USA

Contact
# Correspondence: jroelofs@kumc.edu

Table S1: Strain List

Strain	Genotype (*lys2-801 leu2-3, 2-112 ura3-52 his3-□200 trp1-1)*	Figure	Source
SUB61	MATα		(1)
SUB62	MATA		(1)
sJR288	MATA *rpn4::G418*	**3b**	(3)
sJR622	MATA *scl1::SCL1-CFP (HYG) pre1::pre1-proA (HIS3)*	**3d**	(3)
sJR632	MATα *pre8::PRE8-CFP (HYG) pre1::pre1-proA (HIS3)*	**3d**	(3)
sJR797	MATα *pup1::PUP1-YFP (G418) pre1::pre1-proA (HIS3)*	**3d**	(3)
sJR858	MATα *pre2::PRE2-GFP (HIS3)*	**1c,d 3b,c,e 4c S1d**	(2)
sJR859	MATα *pre2::PRE2-GFP (HIS3) hsm3::G418 nas2::CloNat*	**3e**	(3)
sJR861	MATα *rpn1::RPN1-GFP (HIS3)*	**3b,e S1d**	(2)
sJR862	MATα rpn1*::RPN1-GFP (HIS3) hsm3::G418 nas2::CloNat*	**3e**	(3)
sJR880	MATα *pre2::PRE2-GFP (HIS3) ubp3::HYG*	**1c,d**	(2)
sJR925	MATα *pre2::PRE2-GFP (HIS3) atg7::CloNat*	**1a,e 4a,c,d**	(2)
sJR964	MATα *pre2::PRE2-GFP (HIS3) rpn1::RPN!-mCherry (G418)*	**S1d**	(3)
sJR1053	MATα *pre2::PRE2-GFP (HIS3) hsp42::HYG*	**4c**	(3)
sJR1054	MATα *pre2::PRE2-GFP (HIS3) atg7::CloNat hsp42::HYG*	**4c**	(3)
sJR1084	MATα *scl1::SCL1-GFP (HIS3)*	**1c,d 3c**	(3)
sJR1085	MATα *pre5::PRE5-GFP (HIS3)*	**1c,d 3c**	(3)
sJR1086	MATA *atg7::CloNat scl1::SCL1-GFP (HIS3)*	**1a S1b,d**	(3)
sJR1087	MATA *atg7::CloNat pre5::PRE5-GFP (HIS3)*	**1a S1d**	(3)
sJR1088	MATα *scl1::SCL1-GFP (HIS3) ubp3::HYG*	**1c,d**	(3)
sJR1089	MATα *pre5::PRE5-GFP (HIS3) ubp3::HYG*	**1c,d**	(3)
sJR1092	MATA *atg7::CloNat pre2::PRE2-mCherry (G418))*	**S1b,d**	(3)
sJR1093	MATA *atg7::CloNat pre2::PRE2-mCherry (G418)) scl1::SCL1-GFP (HIS3)*	**S1b,d**	(3)
sJR1103	MATα *pre2::PRE2-GFP (HIS3) rpn11::RPN11-proA (HIS3)*	**2a,b,c**	(3)
sJR1105	MATα *rpn1::RPN1-GFP (HIS3) rpn11::RPN11-proA (HIS3)*	**2a,b,c**	(3)
sJR1155	MATα *pre6::PRE6-GFP (HIS3)*	**3c S1d**	(3)
sJR1160	MATα *pre2::PRE2-GFP (HIS3) atg7::CloNat snx4::G418*	**4d**	(3)
sJR1197	MATA *rpn4::G418 pre2::PRE2-GFP (HIS3)*	**3a,b,c**	(3)
sJR1200	MATA *atg7::CloNat pre2::PRE2-mCherry (G418)* ura3 ^{P}Atg8-*GFP-atg8 (URA3)*	**4b**	(3)
sJR1326	MATA *rpn4::G418 scl1::SCL1-GFP (HIS3)*	**3c**	(3)
sJR1422	MATα *pre2::PRE2-GFP (HIS3) atg7::CloNat rpn1::RPN1-mCherry (G418)*	**1b**	(3)
sJR1423	MATA *atg7::CloNat scl1::SCL1-GFP (HIS3) rpn1::RPN1-mCherry (G418)*	**1b**	(3)
sJR1424	MATA *atg7::CloNat pre5::PRE5-GFP (HIS3) rpn1::RPN1-mCherry (G418)*	**1b**	(3)
sJR1695	MATA *atg7::CloNat pre3::PRE3-GFP (HIS3)*	**S1a,d**	(3)
sJR1696	MATA *atg7::CloNat pup1::PUP1-GFP (HIS3)*	**S1a,d**	(3)
sJR1697	MATA *atg7::CloNat pre1::PRE1-GFP (HIS3)*	**S1a,d**	(3)
sJR1698	MATα *atg7::CloNat pre2::PRE2-GFP (HIS3) rpn2::RPN2-mCHerry (G418)*	**S1b,d**	(3)
sJR1699	MATA *atg7::CloNat rpn5::RPN5-GFP (HIS3)*	**S1b,d**	(3)
sJR1700	MATA *atg7::CloNat pre2::PRE2-mCherry (G418)) rpn5::RPN5-GFP (HIS3)*	**S1b,d**	(3)
sJR1701	MATA *atg7:::CloNat rpn2::RPN2-mCHerry (G418)*	**S1b,d**	(3)

a) All strains have the DF5 background genotype (lys2-801 leu2-3, 2-112 ura3-52 his3-Δ200 trp1-1)

1. Finley, D., Ozkaynak, E., and Varshavsky, A. (1987) Cell 48, 1035-1046
2. Waite, K.A., De La Mota-Peynado, A., Vontz, G., and Roelofs, J. (2015) JBC M115.699124
3. This study

Table S2: Primer List

Primer	Genotype	Template	Sequence (5' to 3')
rvrs/rpn1	*rpn1::rpn1-mCherry (G418)*	pBS34[3]	TTTGAATTTTTCCTATTCTGGTTGATATTGCCCAAAAGCTATTCAGTTTAATCGATGAATTCGAGCTCG
pRL12	*scl1::SCL1-CFP (HYG)*	pBS4	TGCTGAGAACATCGAAGAAAGGCTAGTAGCAATTGCTGAACAAGATGGTCGACGGATCCCCGGG
pRL13	*scl1::SCL1-CFP (HYG)*	pBS4	GTGTTGACGCGTGTGATTTCACATTATGTTGTGGCAGGAAG ATCGATGAATTCGAGCTCG
pRL17	*Pre8::PRE8-CFP (HYG)*	pBS4	GAAAATTAACTTCTCAAGAAATAAATGATAGATTAGAAGCATTAGGTCGACGGATCCCCGGG
pRL18	*Pre8::PRE8-CFP (HYG)*	pBS4	CTTTTGGATAAGTTGAGTGAGATGGGTGATTGGCGGGGATAAT ATCGATGAATTCGAGCTCG
pRL36	*scl1::SCL1-GFP (HIS3)*	pYM28[2]	GTGTTGACGCGTGTGATTTCACATTATGTTGTGGCAGGAAGATCGATGAATTCGAGCTCG
pRL73	*pup1::PUP1-CFP (HYG)*	pBS4	AATATTTGTGACATACAAGAAGAACAAGTCGATATAACGGCT GGTCGACGGATCCCCGGG
pRL74	*pup1::PUP1-CFP (HYG)*	pBS4	TGATTTACTATACTAAAATATACTTAAGTTCTATGTTTTACT ATCGATGAATTCGAGCTCG
pRL236	*atg7::CloNAT*	pAG25[25]	TTCATTATATTTCAACAAATATAAGATAATCAAGAATAAACGTACGCTGCAGGTCGACG
pRL237	*atg7::CloNAT*	pAG25[25]	CGGAAAGTGGCACCACAATATGTACCAATGCTATTATATGCAATCGATGAATTCGAGCTCG
pRL281	*ubp3::HYG*	pFA6a-hphNT1[2]	CCATCATCCAGGTACCGCTTTCCTTTCCATCATCATTAAACGTACGCTGCAGGTCGAC
pRL282	*ubp3::HYG*	pFA6a-hphNT1[2]	TTATTTTTTATGTATTTTGTCTATAATACCACCCCCCGTCATCGATGAATTCGAGCTCG
pRL401	*rpn1::rpn1-mCherry (G418)*	pBS34[3]	AGTAATTTTAAAGAAGAACCCTGACTATCGTGAAGAGGAGGGTCGACGGATCCCCGGG
pRL554	*hsp42::HYG*	pFA6a-hphNT1[2]	CCATATCCCACACAAATTAAGATCATACCAAGCCGAAGCACGTACGCTGCAGGTCGAC
pRL555	*hsp42::HYG*	pFA6a-hphNT1[2]	AATATAAATGTATGTATGTGTGTATAAACAGATACGATATATCGATGAATTCGAGCTCG
pRL589	*scl1::SCL1-GFP (HIS3)*	pYM28[2]	TGCTGAGAACATCGAAGAAAGGCTAGTAGCAATTGCTGAACAAGATCGTACGCTGCAGGTCGAC
pRL590	*pre5::PRE5-GFP (HIS3)*	pYM28[2]	CTTTTACAATTTACGATGGAGAAGCTGTTGCTAAATACATC CGTACGCTGCAGGTCGAC
pRL591	*pre5::PRE5-GFP (HIS3)*	pYM28[2]	CTTTGACAATTGCTTAGTGGAAACGATACTTTGTGTCCAG ATCGATGAATTCGAGCTCG
pRL596	*snx4::G418*	pFA6a-kanMX6	ATTTACGGTATACCACAATACTGCTCTTTTTGTTGAGGATCGTACGCTGCAGGTCGACG
pRL597	*snx4::G418*	pFA6a-kanMX6	AGGTATTATCAGTAGTAATGGGAAAACATTAAGAGCACCAATCGATGAATTCGAGCTCG
pRL602	*pre6::PRE6-GFP (HIS3)*	pYM28[2]	AGAGCAGCAAGAGCAGGACAAAAAGAAAAAATCTAACCAT CGTACGCTGCAGGTCGAC
pRL603	*pre6::PRE6-GFP (HIS3)*	pYM28[2]	TATTTTATATAGGTTTTATGCCCAATATATATCGCCGTTTATCGATGAATTCGAGCTCG
pRL921	*Rpn5::Rpn5-GFP (HIS3)*	pYM28	AATTACAAAAGAGGAAATCATGCACGGTTTGCAAGCTAAACGTACGCTGCAGGTCGAC
pRL922	*Rpn5::Rpn5-GFP (HIS3)*	pYM28	CCGGATCTGAGATAATCCGACACTTACTCGAAAATCTCTATCGATGAATTCGAGCTCG
pRL1004	*pre3::PRE3-GFP (HIS3)*	pYM28[2]	GGAACGTTTGATATTCTACCCTGATGAATATGAACAACTACGTACGCTGCAGGTCGAC
pRL1005	*pre3::PRE3-GFP (HIS3)*	pYM28[2]	AAAGAGAAGTAATCACTCAGAATTTGTTCTTTTGGGGTCAGATCGATGAATTCGAGCTCG
pRL1006	*pup1::PUP1-GFP (HIS3)*	pYM28[2]	TATTTGTGACATACAAGAAGAACAAGTCGATATAACGGCtCGTACGCTGCAGGTCGAC
pRL1007	*pup1::PUP1-GFP (HIS3)*	pYM28[2]	ATTTACTATACTAAAATATACTTAAGTTCTATGTTTTACAGATCGATGAATTCGAGCTCG
pRL1008	*pre1::PRE1-GFP (HIS3)*	pYM28[2]	TAAAGATGGCATAAGACAAGTAGATGACTTCCAGGCACAGCGTACGCTGCAGGTCGAC
pRL1009	*pre1::PRE1-GFP (HIS3)*	pYM28[2]	CATTAGCAATCACCTTTTCCGTGTGATTACACTGAATATCAGATCGATGAATTCGAGCTCG
pRL1012	*Rpn2::RPN2-mCherry (G418)*	pBS34	CCCATTTAAGGTCGATGATAATGTCGACTTCCCTAGTGCTGGTCGACGGATCCCCGGG
pRL1013	*Rpn2::RPN2-mCherry (G418)*	pBS34	GAACTAATTCTTCATTTTCTGTACGACCATGCACCCTTG A TCG ATG AAT TCG AGC TCG

1. Goldstein, A. L. & McCusker, J. H. Three new dominant drug resistance cassettes for gene disruption in Saccharomyces cerevisiae. Yeast 15, 1541–53 (1999).
2. Janke, C. et al. A versatile toolbox for PCR-based tagging of yeast genes: new fluorescent proteins, more markers and promoter substitution cassettes. Yeast 21, 947–62 (2004).
3. Hailey DW, Davis TN, Muller EG. Fluorescence resonance energy transfer using color variants of green fluorescent protein. Methods Enzymol. 351:34-49 (2002.

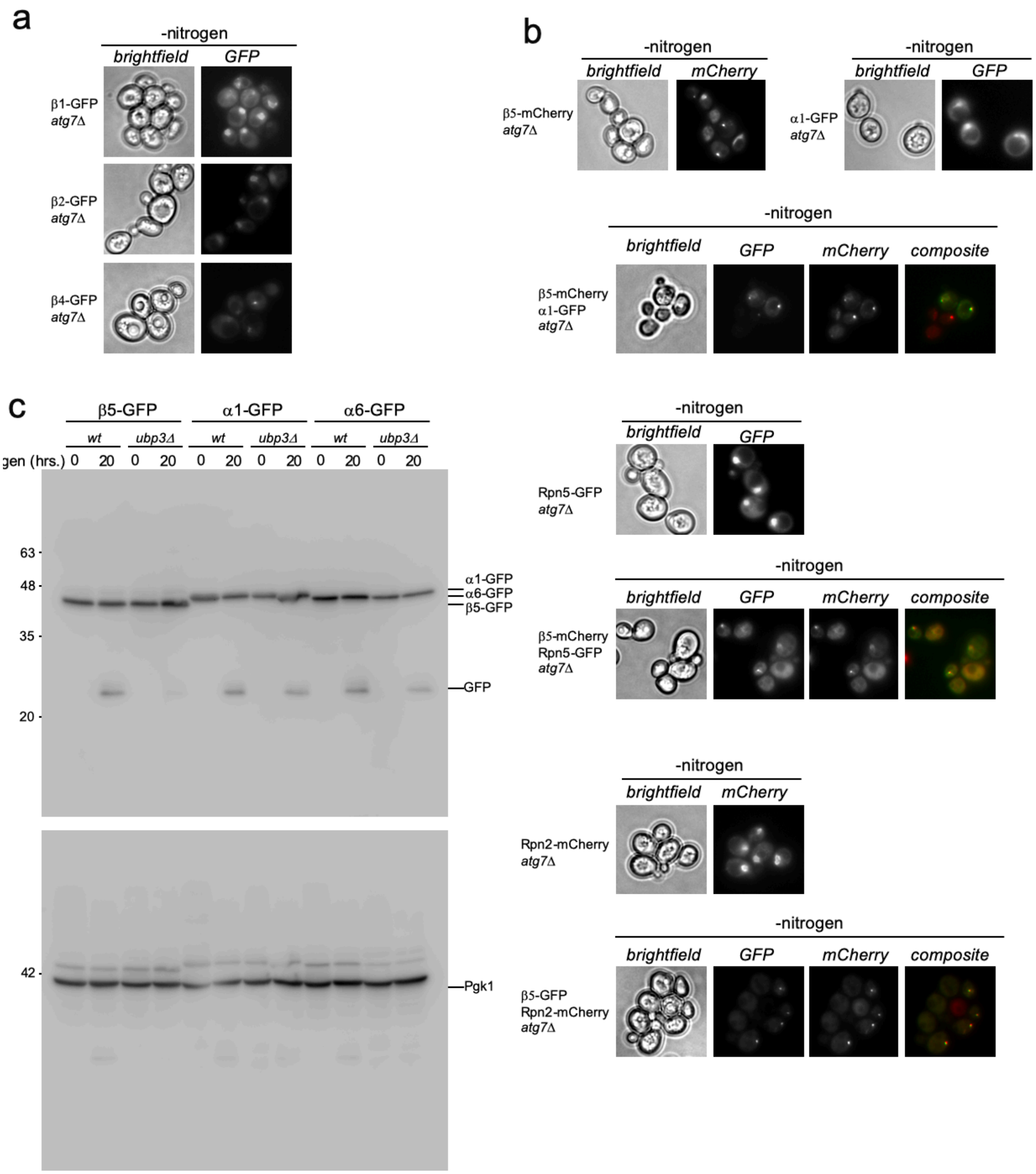

Supplementary Figure S1. Analysis of singly and doubly tagged proteasome following nitrogen starvation (A) *atg7Δ* strains expressing β1-GFP, β 2-GFP or β 4-GFP were starved for nitrogen for 24 hours and analyzed by fluorescent microscopy. **(B)** Indicated singly and double tagged strains were analyzed by fluorescent microscopy following 24 hours of nitrogen starvation. Results show that tagging β5 causes a change in cellular localization of Rpn2-mCherry, as well as Rpn5-GFP and α1-GFP, from nuclei to cytosolic granules. **(C)** Uncropped western blots from Fig. 1C in main text.

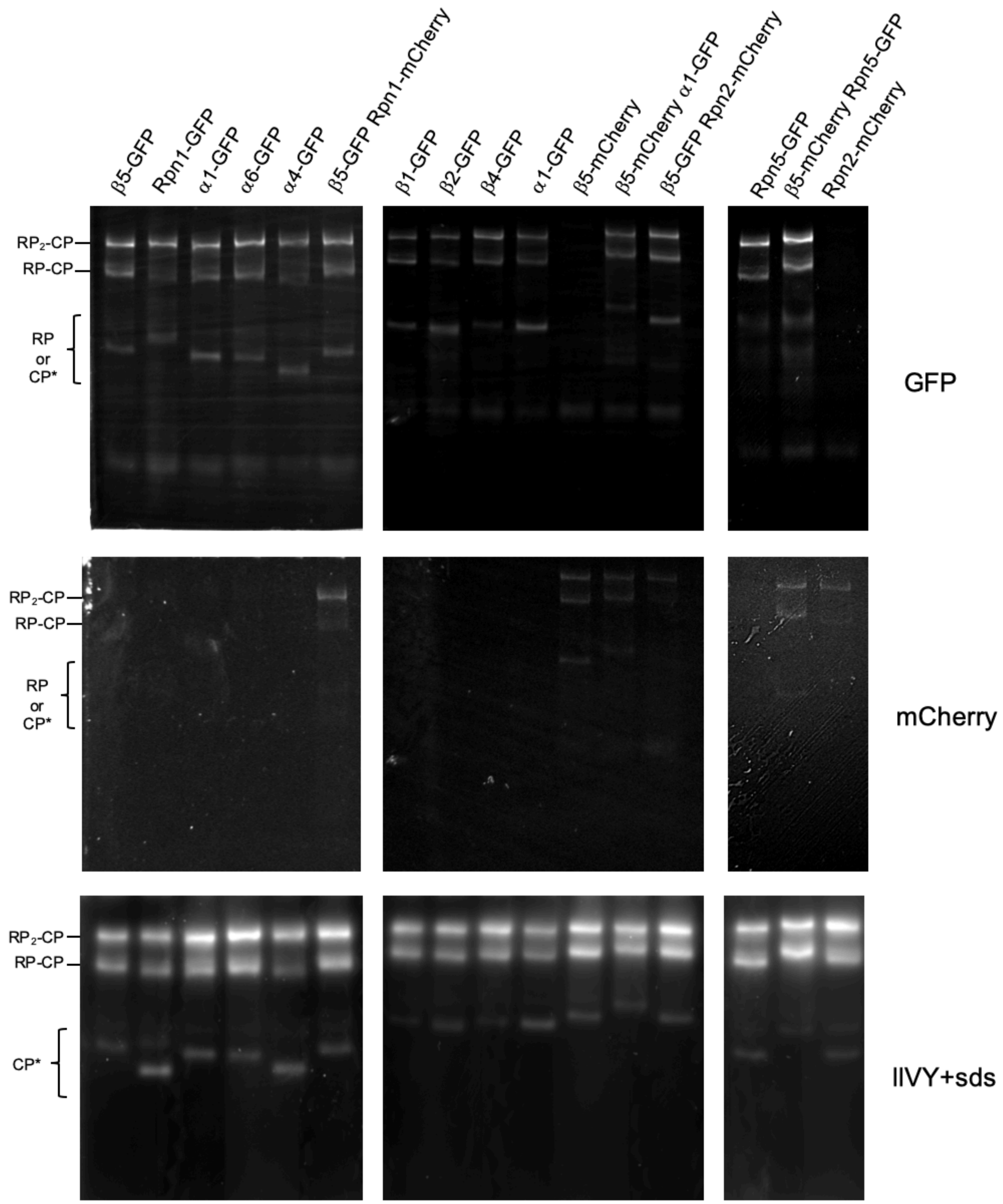

Supplementary Figure S2. Native gel analysis of yeast strains expressing indicated fluorescent tags. Cells were grown overnight in YPD media before cryogrinding pellets to produce lysate as described in materials and methods. Equal lysate was loaded on native gels. Top panels show GFP fluorescence in gel, middle panels mCherry fluorescence, and lower panels suc-LLVY-amc peptidase activity in the presence of 0.02% SDS (opens the CP gate).

Asterix indicates various shifted CPs due to the fluorescent tag. Note that difference in migration between samples on same gel (within each columns) is caused by different retardation on native gel depending on which subunit(s) are tagged. Differences in migration between gels results from gels ran at different times and does not necessarily reflect true differences.

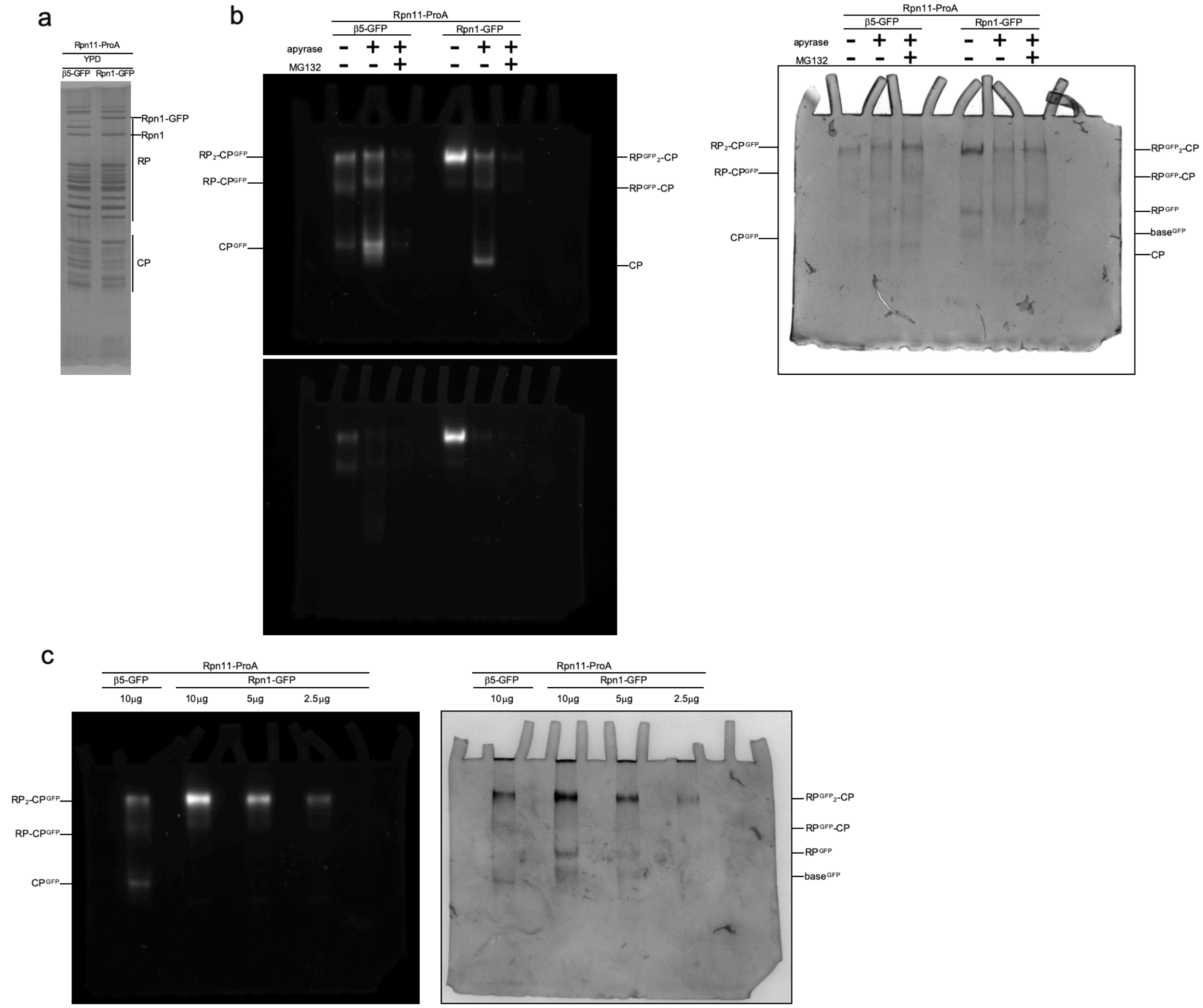

Supplementary Figure S3. (A) Uncropped gels from Fig. 2A, B and C.

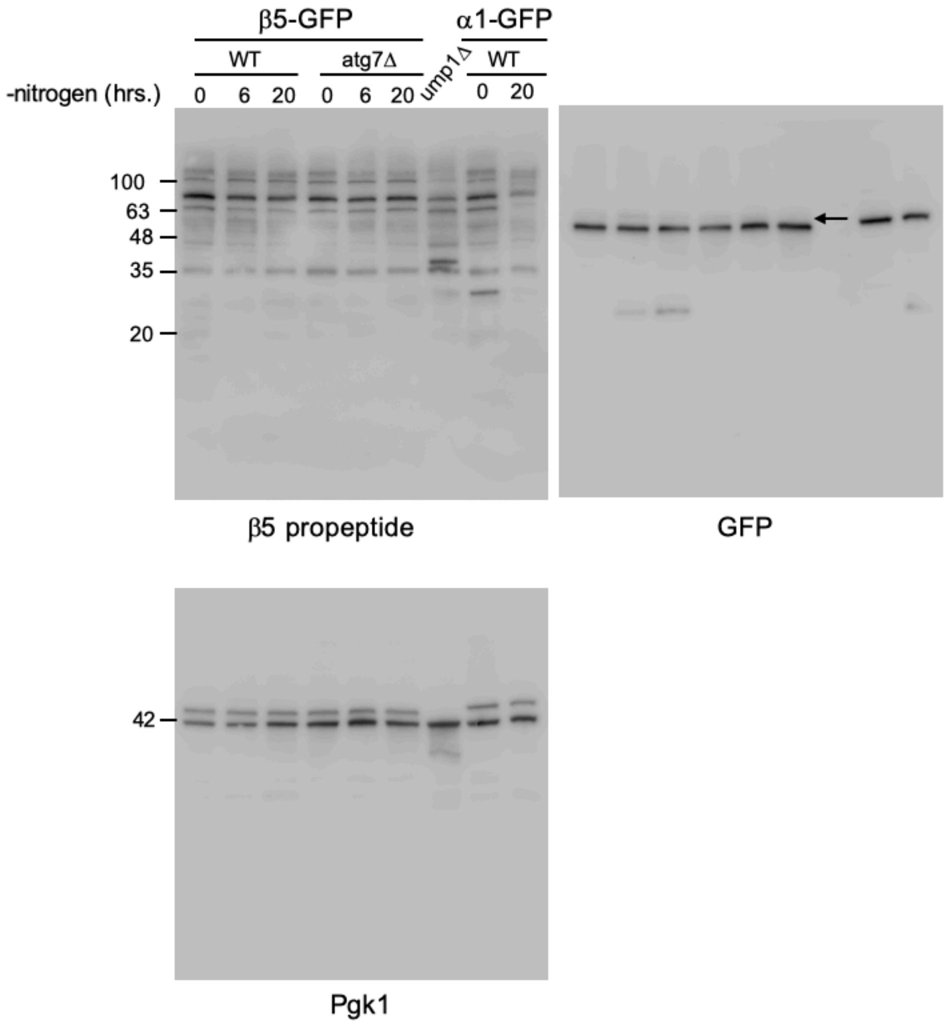

Supplementary Figure S4. β5-propetide analysis following nitrogen starvation. Wild type and *atg7Δ* cells expressing β5-GFP and wildtype α1-GFP cells were starved for nitrogen for the indicated times. Cells were lysed as described in materials and methods, and western blotting for β5-propeptide, GFP and Pgk1 was performed. *ump1Δ* cells were used as a control as these strains accumulate β5-propeptide. Black arrow indicates bands that migrate higher than β5-GFP following nitrogen starvation.
